# Incorporation of Spike and Membrane Glycoproteins into Coronavirus Virions

**DOI:** 10.3390/v7041700

**Published:** 2015-04-03

**Authors:** Makoto Ujike, Fumihiro Taguchi

**Affiliations:** Laboratory of Virology and Viral Infections, Faculty of Veterinary Medicine, Nippon Veterinary and Life Science University, 1-7-1 Kyonan-cho, Musashino, Tokyo 180-8602, Japan; E-Mail: ftaguchi@nvlu.ac.jp

**Keywords:** coronavirus, membrane protein, spike protein, assembly, protein trafficking, intracellular retention signal, protein interactions

## Abstract

The envelopes of coronaviruses (CoVs) contain primarily three proteins; the two major glycoproteins spike (S) and membrane (M), and envelope (E), a non-glycosylated protein. Unlike other enveloped viruses, CoVs bud and assemble at the endoplasmic reticulum (ER)-Golgi intermediate compartment (ERGIC). For efficient virion assembly, these proteins must be targeted to the budding site and to interact with each other or the ribonucleoprotein. Thus, the efficient incorporation of viral envelope proteins into CoV virions depends on protein trafficking and protein–protein interactions near the ERGIC. The goal of this review is to summarize recent findings on the mechanism of incorporation of the M and S glycoproteins into the CoV virion, focusing on protein trafficking and protein–protein interactions.

## 1. Introduction

Coronaviruses (CoVs) are enveloped, positive stranded RNA viruses that cause respiratory, gastrointestinal, hepatic and neurological diseases in mammalian and avian species. Human CoVs (hCoVs) such as 229E, OC43, NL63, and HKU-1 cause ~20% of common cold cases [1,2,3,4,5,6]. Two highly pathogenic hCoVs, Middle East respiratory syndrome coronavirus (MERS-CoV), which were identified in the Middle East and Europe in 2012, and the Severe acute respiratory syndrome coronavirus (SCoV), which emerged in China in 2002–2003, causes severe acute pneumonia and respiratory failure, with 10% and 36% (as of 11 November 2014) fatality rates, respectively [7,8,9,10,11,12]. In the veterinary field, several animal CoVs are known to cause life-threatening diseases. Transmissible gastroenteritis virus (TGEV) and Porcine epidemic diarrhea virus (PEDV), which caused the first outbreak in the United States in 2013, lead to lethal watery diarrhea and dehydration in piglets [13,14,15]. Avian infectious bronchitis virus (IBV) causes infectious bronchitis, a highly contagious respiratory infection in chickens, resulting in reduced meat and egg production [16]. Both TGEV and IBV are on the OIE list of internationally important pathogens [17]. These CoVs are a potential threat to human and animal health, and can cause huge economic losses. 

**Figure 1:**
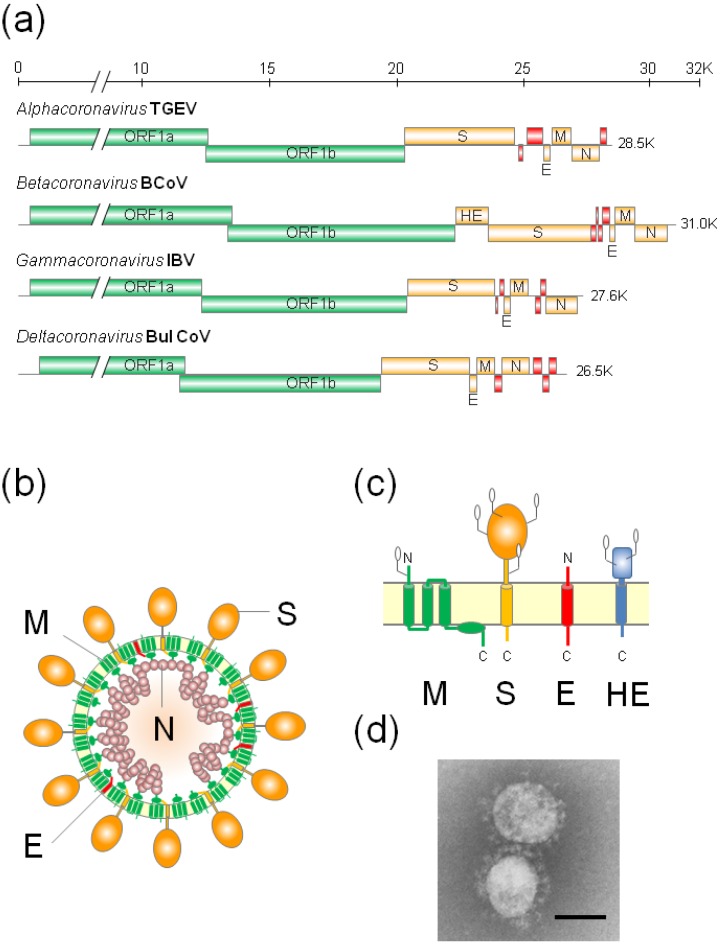
(**a**) Genome organizations of various Coronavirus genera. The first two-thirds of the genome contains open reading frame (ORF)1a and ORF1b, which encode the replicase/transcriptase proteins (Green). The remaining one-third of the genome encodes four structural proteins: spike (S), envelope (E), membrane (M), and nucleocapsid (N) proteins (Orange). Some *betaoronaviruses* have an additional hemagglutinin-esterase (HE) gene (Orange). The genome of each genus or species has a set of unique accessory proteins (red); (**b**) Schematic diagrams of coronavirus virions; (**c**) The topology of the four structural envelope proteins. All proteins are depicted as monomers, but the S and HE proteins form homotrimers and homodimers, respectively. Oligosaccharides are shown on the M, S, and HE proteins. Although a number are omitted, the S and HE proteins contain 21 to 35 and 9 (BCoV HE) potential *N*-glycosylation sites, respectively; (**d**) Electron micrograph of SCoV. Bar: 100 nm. (EM image courtesy of Dr. Nagata at National Institute of Infectious Diseases).

CoVs belong to the order **Nidovirales** and family **Coronaviridae**, and can be divided into four genera: the *alpha-*, *beta-*, *gamma-*, and *deltacoronaviruses* (Table 1) [18,19]. They contain the largest, single-stranded, positive-sense RNA genomes of 26–32 kb, which consist primarily of six conserved open reading frames (ORFs) (Figure 1a) [20,21]. The first two-thirds of the genome contains ORF1a and ORF1b, encoding replicase-transcriptase proteins. These are synthesized as two large polyproteins; pp1a is translated from ORF1a and pp1ab from ORF1a/1b by a programmed ribosomal frameshifting [22]. These polyproteins are proteolytically cleaved into 15 or 16 non-structural proteins [23]. The remaining one-third of the genome encodes four structural proteins: spike (S), envelope (E), membrane (M), and nucleocapsid (N), and a set of strain-specific accessory proteins [24]. Some *betacoronaviruses* contain an additional membrane protein, a hemagglutinin-esterase (HE) [25].

**Table 1:** Coronavirus genus, species, and virus abbreviations.

Genus	Species
**Alphacoronavirus**	Feline Coronavirus (FCoV)
	Transmissible Gastroenteritis Virus (TGEV)
	Porcine Epidemic Diarrhea Coronavirus (PEDV)
	Human Coronavirus 229E
	Human Coronavirus NL63
**Betacoronavirus**	Bovine Coronavirus (BCoV)
	Mouse Hepatitis Virus (MHV)
	Human Coronavirus OC43
	Human Coronavirus HKU-1
	Severe Acute Respiratory Syndrome Coronavirus (SCoV)
	Middle East respiratory Syndrome Coronavirus (MERS-CoV)
**Gammacoronavirus**	Infectious Bronchitis Virus (IBV)
**Deltacoronavirus**	Bulbul Coronavirus HKU11

CoV virions are enveloped and consist of four structural proteins (Figure 1b). The RNA genome is encapsidated by the N proteins into a helical nucleocapsid, surrounded by a lipid envelope. Two major glycoproteins, M protein, which has three transmembrane (TM) domains, and S protein, which has a single TM domain; and minor non-glycosylated E proteins with a single hydrophobic domain, are incorporated into the CoV envelope (Figure 1c). The M and E proteins play important roles in virus morphogenesis, assembly and budding [26,27,28,29,30], while the S protein is responsible for receptor binding and membrane fusion during viral entry [31,32,33]. The S proteins are responsible for the corona (crown-like) projections on the virion surface (Figure 1d). Some *betacoronaviruses* have glycosylated HE proteins with a single TM domain in their envelope. The HE proteins were reported to be involved in the fitness of natural hosts and the production of infectious viruses, although their actual role remains elusive [25,34]. 

**Figure 2:**
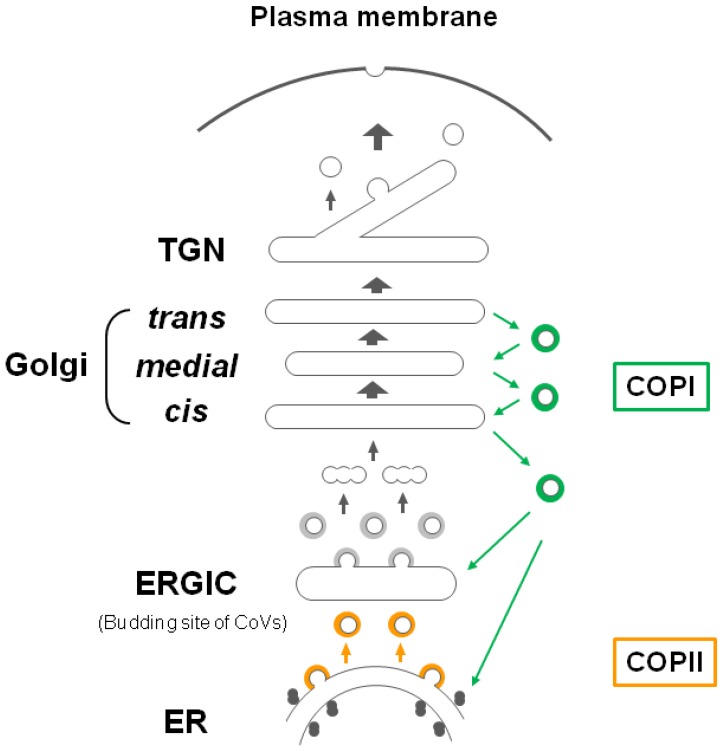
Cisternal maturation and stable (ER)-Golgi intermediate compartment (ERGIC) model. The protein in COPII vesicles (Orange) buds from ER-exit sites to the ERGIC, which is a stable compartment in mammalian cells, and subsequently to the *cis*-Golgi via a second undefined vesicular transport system (Grey) [53,61]. These vesicles fuse homotypically to construct new *cis*-cisternae, which are not stable compartments. They move and mature from *cis* to *trans*, and then breakdown into transport carriers at the TGN. Transport between the ER and Golgi is controlled by COPI vesicles (Green) in retrograde, and COPIl vesicles in anterograde, transport [55,56,58]. (Figure is modified, based on figure of Glick *et al.*, reference 56).

One characteristic of CoVs is virion assembly at and budding into the lumen of the endoplasmic reticulum (ER)-Golgi intermediate compartment (ERGIC) (Figure 2), followed by release by exocytosis [35,36,37]. For most enveloped viruses, this process occurs at the plasma membrane. For efficient CoV virion assembly, three membrane (enveloped) proteins must be retained near the intracellular compartment ERGIC, as membrane proteins generally reach the plasma membrane through the secretory pathway. In fact, the M, E and some S proteins contain intracellular trafficking signals that result in their targeting to, and accumulation near, the budding site [38,39,40]. In addition, protein-protein interactions (as well as protein-RNA interactions) are important for efficient virion assembly. M proteins play a critical role in this function since virus-like particle (VLP) formation in many CoVs requires only the M and E proteins [26,30,39], but formation of the SCoV VLP is controversial and may require M/E [41], M/N [42], M/N/E [43], or only M [44] proteins. In any cases, the M protein is essential, and homotypic M-M interactions through multiple contact sites are required to drive VLP and CoV assembly [45,46]. In addition, incorporation of E, S, and ribonucleoproteins (RNPs) into virions is mediated by heterotypic interactions with M proteins at the budding site [47,48,49,50,51,52]. Thus, the efficient incorporation of viral proteins into CoV virions depends on two important determinants: protein trafficking to, and protein–protein interactions at, the ERGIC. In this review, we summarize recent findings on the mechanism of incorporation of the major M and S glycoproteins into CoV virions, focusing on the abovementioned two important determinants. 

## 2. The Glycoprotein Trafficking and Intracellular Retention Mechanism

We summarize here the many excellent reviews on protein trafficking through the secretory pathway, from the ER to the plasma membrane though the Golgi complex [53,54,55,56,57,58], and on the mechanism of intracellular retention [59,60]. Figure 2 shows a schematic of this trafficking pathway. 

Newly synthesized proteins, such as M, S and E proteins, enter the ER where they are folded and packaged into vesicles formed by coat protein complex II (COPII), which bud from specific ER-exit sites (Figure 2) [56]. CoVs bud and assemble in the ERGIC between the ER and the Golgi complex. Previously, ERGIC, which is formed by fusion of COPII vesicles, was regarded as a transient mobile compartment from the ER to the Golgi complex. However, recent live cell imaging suggests that the ERGIC is a stable, stationary compartment in mammalian cells [53,61]. 

In this model, transport from the ER to the ERGIC is mediated by COPII vesicles. Subsequent transport to the *cis*-Golgi is mediated by a second vesicular transport system. Proteins from the ERGIC pass thorough the Golgi complex, which can be divided into four compartments; *cis*, *medial*, and *trans*-cisternae, and the *trans-*Golgi network (TGN), during which they are modified by glycosylation. Although the protein pathway within the Golgi complex has been debated, much of the data support the Golgi cisternal maturation model [55,56,58]. In this model, the Golgi cisterna is not a long-lived, stationary entity but a transient compartment. Vesicles budding from the ERGIC fuse homotypically to construct new *cis*-cisternae, move and mature from *cis* to *trans-*cisternae, and then break down into transport carriers at the TGN. Resident Golgi proteins (e.g., glycosylation enzyme) or some proteins can return to the ER or the younger cisternae by retrograde transport. This retrograde transport is mediated by COPI vesicles, which function primarily in recycling proteins from the Golgi complex to the ER (Figure 2) [54,55,56]. Thus, retrograde transport between the ER and the Golgi complex is controlled by COPI vesicles; COPII vesicles mediate anterograde transport. Proteins at the TGN are transported either directly to the plasma membrane or indirectly via recycling endosomes [57]. 

The intracellular retention mechanisms differ between the ER and Golgi complex. Proteins resident in the ER typically contain two well-known retrieval signals: the KDEL motif of secretory proteins and the KKxx motif of membrane proteins. Membrane proteins with the KKxx motif in their cytoplasmic tail (CT) domain bind directly to COPI and are packaged into COPI vesicles, and then returned to the ER via retrograde transport, resulting in retention in the ER [59]. In contrast, no common signal for retention in the Golgi complex has been identified, although many mechanisms of Golgi complex retention have been reported [60]. One of these mechanisms is the kin-recognition model. It is proposed that resident Golgi proteins form a large hetero-oligomer at certain cisternae, and these aggregated protein clusters are thought to be too large to be physically included into transported vesicles, resulting in retention in the Golgi complex [62,63]. 

In the case of CoVs, the E, M, and some S proteins have been reported to be retained in the ERGIC/Golgi-complex at a steady state. It is important to note that the retention of proteins in the intracellular compartments at steady state is a highly dynamic process, requiring iterative rounds of retrograde (Golgi–ER) and anterograde (ER–Golgi) transport. 

## 3. M Proteins

### 3.1. General Properties

**Figure 3:**
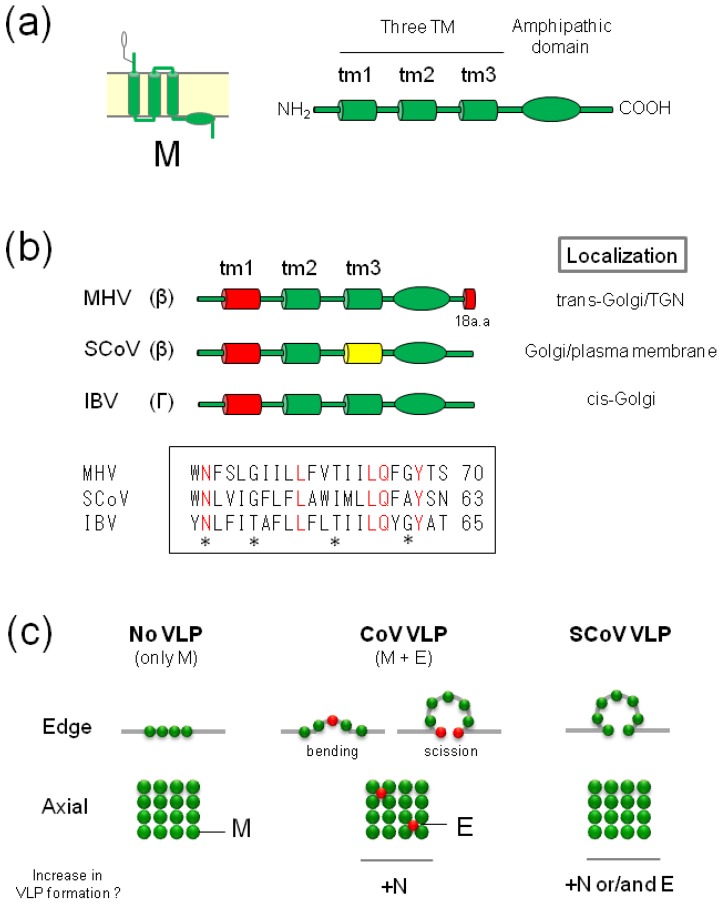

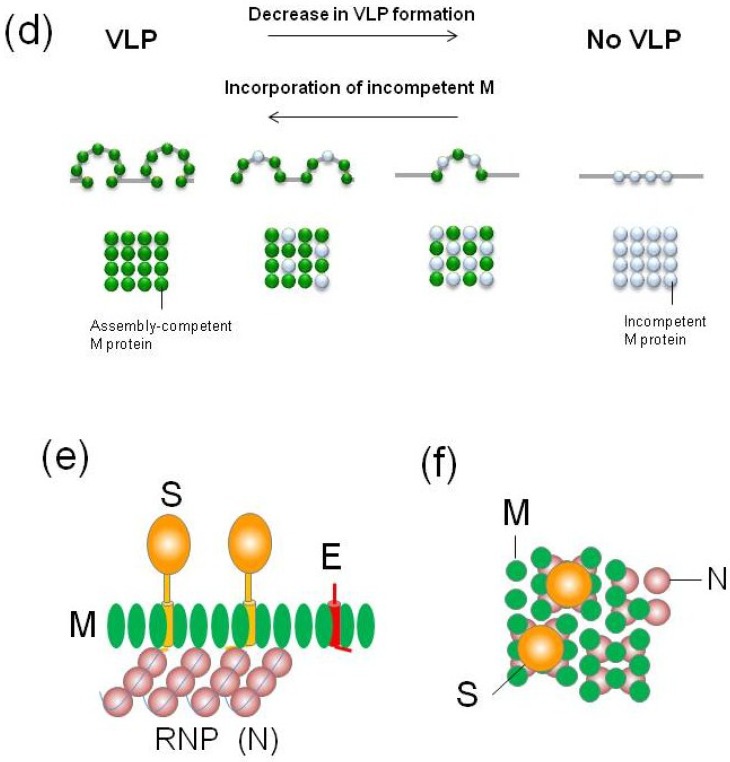
(**a**) Topology and schematic diagram of coronaviruses (CoV) M proteins. Three TM domains were assigned to tm1, tm2, and tm3 regions; (**b**) Trafficking signals of CoV-M proteins. Red box shows the identified intracellular retention signal, and the yellow box the plasma membrane targeting signal. The tm1 regions of SCoV- and infectious bronchitis virus (IBV), but not MHV-, M proteins are sufficient for intracellular retention (Top). Amino acid sequences of the tm1 regions of three CoV-M protein (Bottom). Conserved amino acids are shown in red. Asterisks indicate the uncharged polar residues critical for intracellular retention of VSV-G with tm1 regions of IBV-M [82]; (**c**) Minimum requirement for virus-like particle (VLP) formation. Coexpression of M and E proteins, but not M protein alone, resulted in formation of VLPs. E proteins might cause membrane bending or scission at the budding site. N protein likely assists VLP formation. In contrast, SCoV-M protein alone resulted in production of VLP, albeit at a low density. The minimum requirement for SCoV VLP assembly is controversial; (**d**) The importance of M-M interactions via multiple contact sites in the overall domain for VLP incorporation. Assembly-incompetent MHV-M proteins (light blue) lacking parts of the TM domain, the amphipathic domain, or the *C*-terminal domain, or M proteins in which the *N*-terminal domain has been replaced by exogenous proteins, lack VLP formation ability. An increase in the quantity of assembly-competent M proteins (green) promotes VLP formation and provides more opportunities for the binding and capture of incompetent M proteins into VLPs, resulting in an increase in the co-incorporation of incompetent M proteins into VLPs or virions [46]. E proteins are omitted; (**e**,**f**) Lattice-like matrix model of M protein in edge (**e**) and axial (**f**) views. M protein would form a lattice-like matrix within the envelope. RNPs would interact with M proteins to be packaged into virions, and S proteins are accommodated within spaces of this lattice via interaction with M proteins. Small numbers of E proteins are inserted into the other spaces within the lattice.

The M protein (~230 amino acids, 22–25 kDa) is the most abundant protein in the viral envelope. Despite relatively low amino acid sequence homology (less than 30%), the overall structure of the M proteins of various CoVs is conserved (Figure 3a). CoV-M protein consists of a short glycosylated *N*-terminal ectodomain, three TM domains (labeled the tm1, tm2, and tm3 regions, from the *N*-terminal), and a long *C*-terminal CT domain. The *C*-terminal CT domain is divided into a closely membrane associated, amphipathic domain following the tm3 region, and a short hydrophobic domain at the tail end (Figure 3a), resulting in the topology of an *N*-terminal ecto- and a *C*-terminal endodomain (Nexo-Cendo) [64,65,66]. One exception is the TGEV-M protein, which exists in two topologies: the Nexo-Cendo orientation and less commonly the Nexo-Cexo-orientation [67,68]. All CoV-M proteins have a glycosylation site in the *N*-terminal ectodomain, which undergoes either *N*-glycosylation in *alpha-* and *gammacoronaviruses* or *O*-glycosylation in *betacoronaviruses* [69,70,71]. It is noteworthy that the glycosylation of CoV-M proteins is not involved in their trafficking or VLP or virion assembly [44,69].

### 3.2. Golgi Retention Signal of CoV-M Proteins

The M proteins of most CoVs accumulate at the ERGIC and predominantly the Golgi complex, and are not detected at the plasma membrane of infected cells or single M-expressing cells [40,72,73,74,75], indicating that most CoV-M proteins contain an intrinsic retention signal that leads to their accumulation in the Golgi complex in the absence of the other viral proteins. Within the Golgi complex, each CoV-M is distributed differently (Figure 3b). The IBV-M protein is localized to the *cis*-Golgi [72,76], while the MHV-M protein is localized to the *trans*-Golgi and the TGN [72,77]. In some cases, however, the TGEV-M protein [78,79] and the feline infectious peritonitis virus (FIPV) M protein [80] in infected cells or independently expressed epitope tagged or untagged SCoV-M protein [44,81] were transported to the plasma membrane. It is noteworthy that, although M proteins play an essential role in the CoV budding process, which occurs exclusively at the ERGIC, a large portion is transported to the Golgi complex or even the plasma membrane beyond the ERGIC [72]. Thus, CoV-M is proposed to recycle from the Golgi-complex to the ER [46] and to the Golgi-complex from the plasma membrane via endocytosis. 

The Golgi retention domains of IBV-M and MHV-M, which accumulate primarily in the Golgi complex, and of SCoV-M, which can reach the plasma membrane, have been investigated extensively. Mutational experiments of IBV-M proteins revealed that the first (tm1) of the three TM domains contains a *cis*-Golgi retention signal [40,76], and that this domain alone is sufficient for retention of the exogenous protein at the *cis*-Golgi (Figure 3b). In contrast, MHV-M proteins require two domains of the tm1 region and 18 amino acids at the *C*-terminal for retention [83,84,85]. Each domain alone, however, is not sufficient for retention of mutant M or exogenous proteins in the Golgi complex [82,83,84,85], indicating that each tm1 region and 18 amino acids of the *C*-terminal domain are essential, but not sufficient for, Golgi retention, and that both domains are required. On the other hand, the SCoV-M protein is localized primarily both at the plasma membrane and the Golgi complex. Deletion of wt M protein fused with a fluorescent protein showed that the tm1 region of SCoV-M contains a Golgi retention signal identical to that of IBV-M. Interestingly, the tm3 region was found to contain a plasma membrane trafficking signal (Figure 3b) [44]. 

Three tm1 regions of CoV-M proteins are clearly involved in Golgi retention. Those of IBV- and SCoV-M are sufficient for Golgi retention alone, but those of MHV-M proteins are not. A comparison of the tm1 regions indicates no distinct differences in composition or length (Figure 3b), suggesting that subtle differences in amino acid sequence have a marked effect on retention, as reported previously [82]. The mechanism underlying the retention of CoV-M in the Golgi is correlated with the oligomerization of M proteins in the Golgi complex [86,87]. VSV-G proteins with the tm1 region of IBV-M (termed Gm1 [86]), which are retained in the Golgi, can form a stable and large oligomer, while Gm1 mutants with substitutions in the tm1 region, which lack Golgi retention ability and are released to the plasma membrane, cannot form such oligomers [86]. Similarly, wt MHV-M proteins form large heterogeneous oligomers in the Golgi complex, whereas MHV-M mutants lacking one or two TM domains show reduced or no oligomerization. In addition, an MHV-M mutant protein lacking *C*-terminal amino acids, which lacks Golgi retention ability and is released to the plasma membrane, can form oligomers; however, these are significantly smaller than wt MHV-M, suggesting that oligomer size is an important factor in Golgi retention [87]. Both findings suggest the importance of M oligomerization in the Golgi complex for retention. However, oligomerization is not the sole determinant of retention, and it is unclear whether large oligomers of MHV-M proteins are comparable to those of Gm1 proteins, since wt IBV-M proteins cannot form heterogeneous oligomers, similar to MHV-M under identical experimental conditions [87]. Several retention mechanisms have been proposed for CoV-M proteins. The structure of the large M oligomer might bind or contain specific lipids or Golgi-resident proteins or interact with components of Golgi subcompartments, thereby facilitating retention. Alternatively, as proposed in the kin recognition model, excessively large oligomers of CoV-M proteins at certain cisternae may not be physically included in transported vesicles, resulting in their retention [86,87].

### 3.3. Incorporation of CoV-M into VLPs or Virions

The importance of the multiple regions and homotypic interactions for VLP or virion assembly has been reported in MHV [45,46,88] and SCoV-M proteins [89]. Since VLPs enable investigation of the mechanism of viral protein incorporation into virions, the VLP incorporation assay is frequently used, but the minimum viral protein requirement for VLP formation differs between MHV and SCoV. Although independently expressed MHV-M proteins form large oligomers in the Golgi complex [87], but are not sufficient for VLP formation, MHV-M proteins facilitate VLP formation in the presence of E proteins (Figure 3c) [26]. Similar observations were reported for co-expression of the M and E but not N proteins of IBV, TGEV and BCoV [30,39], indicating that CoV-E proteins are essential for CoV-M protein incorporation into VLPs. It was noted, however, that although previous experiments used a vaccinia virus-based expression system, a virus-free plasmid-based system was used to demonstrate the involvement of MHV- and IBV-N proteins in VLP formation [90,91]. In contrast, independently expressed SCoV-M proteins undergo oligomerization and are secreted into the culture medium as M-formed vesicles, which are of a slightly lower density than VLPs (Figure 3c) [44]. The minimum requirement for SCoV VLP assembly is controversial. Co-expression of the M and E [41] or M and N [42] proteins results in the production of SCoV VLPs, and M, N, and E proteins are required for efficient VLP formation [43]. Several mechanisms of E or N protein-mediated virion assembly have been postulated. The CT domains of the IBV-E and -M proteins interact with each other, and this may be required for virion assembly [92,93]. The E proteins may insert into the lattice of M proteins [26] and be involved in the induction of membrane bending [51] or in the scission of particles at the budding site (Figure 3c) [29,90]. On the other hand, the role of N proteins is unknown; however, CoV-N proteins predominantly form dimers in solution even in the absence of nucleic acids [94]. Moreover, several studies have suggested that CoV-N proteins can form high-order oligomers [95,96,97,98,99]. *N*-Oligomerization may result in the generation of a scaffold or enhance stability to promote M oligomerization and so facilitate VLP and virion assembly.

The VLP or virion incorporation assay, by co-expression of mutant MHV-M and wt E proteins, showed the importance of overall domains of MHV-M proteins for VLP formation [45]. M mutants lacking the *N*-terminal or TM domain, or the terminal end or amphipathic domain of the *C*-terminal, exhibited impaired VLP formation to some extent. Notably, deletion of only a single amino acid at the *C*-terminal prevented VLP formation, while the complete virus with the same mutation exhibited no such effect, suggesting that another component in the complete virion, most likely the N protein, could compensate for this mutation in the assembly process. However, further truncation of the *C*-terminal end prevented formation of virions as well as VLPs, suggesting that the extreme *C*-terminal end plays an essential role in virion assembly. Recent reports have shown that the 12 conserved amino acids in the amphipathic domain of MHV-M are essential for VLP and virion assembly, while some substitutions in these amino acids inhibited VLP formation but could be rescued into the complete virions, suggesting that the 12 amino-acid is important and that, similarly, another protein, such as the N protein, facilitates the M protein-mediated assembly process [88]. In addition, as described in Figure 3d, assembly incompetent MHV-M proteins with mutations in different domains, which lack the ability to produce VLPs, could interact with other M proteins and be incorporated into VLPs formed by assembly competent M proteins [46]. These observations suggest the importance of M-M interactions via multiple contact sites of overall domains for VLP formation. 

The SCoV VLP incorporation assay involved the co-expression of mutant M and wt N proteins or M/N/E proteins. Deletion of the CT domain and three TM domains resulted in loss of VLP formation [42]. Moreover, single or double amino acid mutations of overall domains indicate the importance of specific amino acids of the M protein for VLP formation, similar to MHV-M proteins [89]. 

These studies indicate that M protein incorporation into virions or VLPs is required for the presence of E (or N) proteins and, moreover, for homotypic M-M interactions though multiple contact sites of its overall domains. Based on these biochemical studies, one hypothesis is that M proteins would form a lattice-like dense matrix due to lateral M-M interactions within the viral envelope [45,46], which is partially observed in morphological studies [100,101]. This lattice-like matrix is proposed to play many roles in virion assembly: (i) provide an assembly scaffold along with the E or N proteins; (ii) package RNPs into virions by interacting with N proteins; (iii) exclude foreign proteins by occupying the space and not interacting with them; and (iv) incorporate S (or HE proteins) into the vacancies of the lattice via specific interactions with M proteins, resulting in regular positioning of these proteins in the lattice (Figure 3e,f). Because of the significantly different space filling between S homotrimers and HE homodimers, there might be two types of vacancies for each protein [45,46].

## 4. S Proteins

### 4.1. General Properties

**Figure 4:**
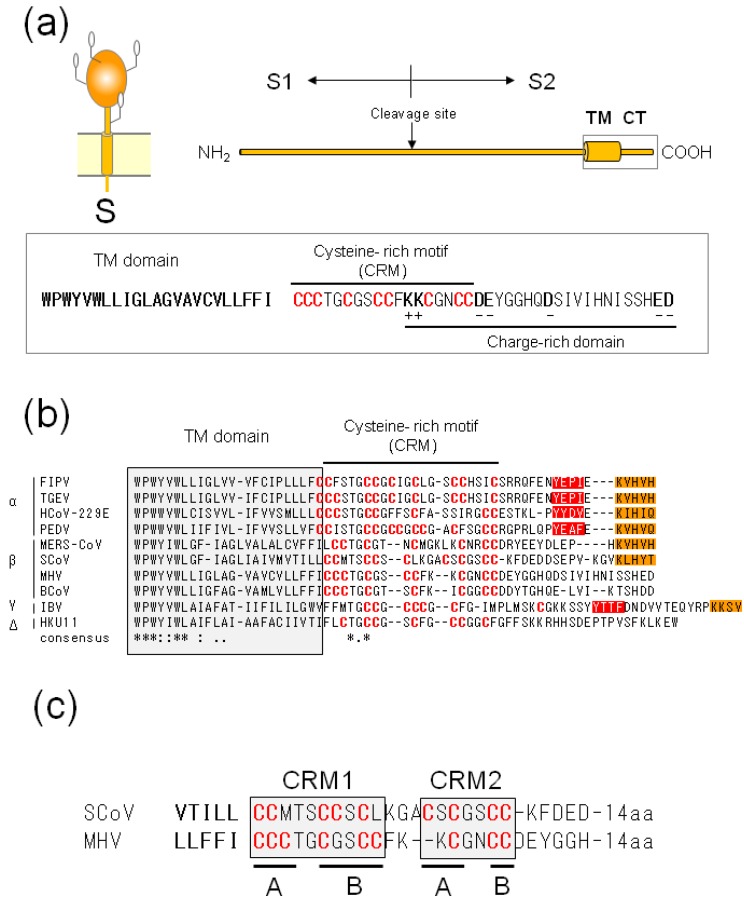
(**a**) Schematic diagram of CoV-S proteins and CT domain amino acid sequences. The CoV S2 domain can be further divided into three subdomains; a large ectodomain, a single TM domain, and a CT domain (top). The deduced CT domain sequence of MHV-S (A59) protein comprises two subdomains: a cysteine-rich motif (CRM) and charge-rich domains, which partially overlap (bottom); (**b**) *C*-terminal ends of 10 CoV-S proteins. Amino acid sequence alignment was performed by CLUSTALW. Shaded box indicates the deduced TM domain. Cysteine residues in CRM are shown in red. Orange and red boxes indicate potential ER retrieval signals (KxHxx- or KKxx-motif) and tyrosine-dependent localization signals/internalization signals (YxxФ motif, where Ф can be F, I, L, M or V), respectively. (**c**) Comparison of the CRM domains of SCoV- and MHV-S. The CRM can be further divided into four subclusters.

The S protein (average 1300 amino acids, 180–200 kDa) forms a trimeric spike on the virion surface. This protein is highly glycosylated, containing 21 to 35 potential *N*-glycosylation sites, and is cleaved into S1 and S2 domains by host or exogenous proteases (Figure 4a) [33,102,103]. The S1 domain contains a binding domain for a host cell receptor, while the S2 domain is responsible for viral entry and cell fusion. The CoV S2 domain can be further divided into three domains; a large ectodomain, a single TM domain, and a CT domain. Although the exact border between the TM and CT domains has not yet been identified, the CT domain may range from 35 (BCoV) to 48 (IBV) amino acids (Figure 4b) [104]. The deduced MHV-S CT domain contains two subdomains: a cysteine-rich motif (CRM) and charge-rich domains, which partially overlap (Figure 4a). Some of the cysteine residues in the CRM appear to be modified with palmitic acid [105,106,107]. The CT domain of some CoV-S protein contains intracellular retention signals and is also important for S protein incorporation into VLPs and virions. 

### 4.2. Intracellular Retention Signal of CoV-S Proteins

Several CoV-S proteins (FIPV, MHV, and IBV) showed slower transport kinetics than general membrane proteins when expressed independently, suggesting the presence of intracellular retention signals [108]. Two such signals have been identified in the CT domain of CoV-S proteins: ER retrieval signals (KKxx- or KxHxx-COOH) and tyrosine-dependent localization signals (YxxI or YxxF motif) [38,109,110,111]. Although the tyrosine-dependent localization signals are identical to the well known YxxФ motif (where Ф can be F, I, L, M or V) that functions as an internalization signal from the cell surface [112,113], the tyrosine-based signal of some CoV-S seems to function as a retention signal instead of as an internalization signal, and vice versa. The efficiency and presence of these signals, however, differs among CoV genera or species (Figure 4b). The S proteins of *alphacoronaviruses*, such as TGEV and PEDV, and the *gammacoronaviruses*, such as IBV, with two signals, are primarily retained intracellularly and rarely detected on the cell surface when expressed independently [38,109,114]. In contrast, the *betacoronavirus* SCoV-S, which contains an ER retrieval signal, and MHV, which has no such signal, are primarily transported to the cell surface. SCoV- and MHV-S proteins, however, when co-expressed with M proteins, were retained near the budding site [49,110]. These observations indicate that the S protein of each CoV genus has inherently different intracellular retention properties. 

Although the CT domains of IBV- and TGEV-S contain two signals, their roles are controversial (Table 2) [38,109,111]. IBV-S and TGEV-S contain a dilysine (KKxx-COOH) or a dibasic (KxHxx-COOH) ER retrieval signal and a potential tyrosine-dependent localization signal (YxxF or YxxI motif), respectively. Independently expressed IBV-S proteins accumulate in intracellular compartments, such as the ER and ERGIC [38] or a late Golgi region [111], and are not detected on the cell surface. One group reported that the dilysine signals of the full IBV-S protein and a chimera between the IBV-S CT domain and the VSV-G ectodomain play an important role in protein accumulation near the budding sites [38]. Moreover, the tyrosine-based signal (YxxF motif) of a chimeric protein functioned as an internalization signal, but not a retention signal [38]. In contrast, the other group reported that the tyrosine-based signal, but not the dilysine signal, of the full IBV-S protein, is the main determinant of intracellular retention [111]; the loss of its internalization function was also demonstrated. A similar discrepancy was observed for TGEV-S. One group reported a tyrosine-based signal (YxxI motif), but not a dibasic signal (KxHxx-COOH) of the full TGEV-S protein, to be the main intracellular signal [109], while another group reported that a chimeric protein with the TGEV-S dibasic signal was retained [38]. The discrepancy might be due to use of different cell types or the fact that one group used mainly chimeric proteins. Recently, the role of the ER retrieval signal of PEDV-S has been reported [114]. The PEDV-S protein contains two signals: a dibasic (KxHxx-COOH) and a potential tyrosine-based (YxxF motif) signal. A large portion of wt PEDV-S proteins was retained intracellularly, whereas S proteins with a mutation of the dibasic signal (H → R) exhibited enhanced cell surface expression, suggesting the involvement of the KxHxx motif of wt PEDV-S in intracellular retention [114], although the role of the YxxF motif remains unclear. 

On the other hand, the CT domain of SCoV-S contains only a dibasic ER retrieval signal (KxHxx-COOH). Although the KxHxx motif of SCoV-S showed a reduced rate of trafficking through the Golgi complex and partially retained exogenous proteins in the ERGIC, both the wt SCoV-S and a mutant S protein lacking the KxHxx motif were detected on the cell surface to the same extent, suggesting that the motif of SCoV-S is less potent than that of PEDV-S [110]. However, when co-expressed with M proteins, wt SCoV-S could be retained at the ERGIC/Golgi complex via an interaction with M, while mutant S is released to the cell surface, suggesting that the KxHxx motif of SCoV-S provides sufficient opportunity to interact with M by recycling between the ER and Golgi complex [110]. These findings indicate that, despite harbouring an identical KxHxx motif, the inherent retention potency differs significantly among CoV species (Table 2).

The above reports indicate the importance of two signals—ER retrieval and tyrosine-based signals—for intracellular retention. The role of ER retrieval signals is well established. As described above, the ER retrieval signal binds directly to COPI proteins, recruiting them into COPI vesicles for trafficking from the Golgi to the ER by retrograde transport. Since the binding affinity to COPI depends on the amino acid sequence surrounding the ER retrieval signal [115], it is conceivable that CoV-S proteins containing the same motif (KxHxx) show different intracellular retention potencies [109,110,114]. On the other hand, although the tyrosine-dependent localization signals (YxxF or YxxI motif) of CoV-S are identical to the well-known tyrosine-containing motif YxxФ that plays an important role in post-Golgi sorting (e.g., rapid internalization), the function is different. Since the tyrosine motif YxxФ must interact with a cellular binding partner to function, identical YxxI or YxxF motifs might bind unknown cellular proteins that mediate intracellular retention. It is important to note that the CT domains of the S proteins from the *betacoronaviruses* MHV and BCoV do not contain both an ER retrieval signal (KKxx or KxHxx motif) and a tyrosine-dependent localization/internalization signal (YxxФ motif) (Figure 4b). *Beta* CoV-S proteins—but not *alfa* and *gamma* CoV-S—lack retention signals and are easily released to the cell surface to promote cell-cell fusion; thus, *betacoronaviruses* seem to use cell-cell fusion positively to disseminate their genes.

**Table 2:** Summary of the roles of the retention signals of several CoV-S proteins.

Genus	Species	Tyrosine-Dependent Localization/Internalization Signals (I.S.)	ER Retrieval Signal
***Alpha* CoV**	**TGEV**	**YxxI motif** **Main retention signal. Not I.S.** [109]	**KxHxx motif** Not main retention signal [109] **Retention of reporter protein** [38]
	**PEDV**	**YxxF motif** Not studied	**KxHxx motif** **Retention signal** [114]
***Beta* CoV**	**SCoV**		**KxHxx motif Weak retention signal** [110]
***Gamma* CoV**	**IBV**	**YxxF motif** **Main retention signal. Not I.S.** [111] I.S. Not main retention signal [38]	**KKxx motif** **Main retention signal** [38] Not main retention signal [111]

### 4.3. Incorporation of CoV-S Proteins into VLPs or Virions

The S protein is dispensable for virion assembly. When infected cells are treated with tunicamycin, a spike-less and noninfectious virion is produced [116,117]. Moreover, CoV VLP formation does not depend on the S protein, but when present the S proteins are incorporated into VLPs [26,30,39,42]. The interaction of S and M proteins is essential for S-incorporation, and a specific M-S interaction has been demonstrated by coimmunoprecipitation assay in infected cells or cells co-expressing the M and S proteins of MHV or BCoV [48,49]. In addition, an immunofluorescence assay showed that independently expressed S proteins are transported to the cell surface, whereas they are retained intracellularly near the budding site and co-localized when co-expressed with M proteins [48,49]. These experiments have resulted in identification of the primary domain of each M and S protein required for the M-S interaction or S-incorporation into VLPs or virions. 

The M protein sequence requirement for M-S interactions has been identified in mutational studies of MHV- and SCoV-M [118,119]. Deletion of the main part or only a single tyrosine at position 211 of the amphipathic domain of MHV-M proteins resulted in no or severely reduced M-S interaction, while mutation of the other domains did not completely inhibit M-S interactions, suggesting that the structural integrity of the amphipathic domain of MHV-M proteins is important for M-S interactions. Similarly, despite its different position in MHV-M, a single tyrosine of SCoV-M at position 195 is essential for M-S interactions [119]. Interestingly, a single tyrosine residue at a specific position in the CT domain of the MHV- and SCoV-S proteins plays a critical role in interactions with S proteins, albeit at different positions. It is thus possible that tyrosine-based post-translational modifications such as phosphorylation and nitrotyrosine are responsible for M-S interactions, although SCoV-M proteins have been reported to not be phosphorylated [119]. Alternatively, as mentioned above, the well-known tyrosine motif YxxФ may bind a cellular binding partner. Since hydrogen bonding via the hydroxyl group of tyrosine plays an essential role in this function [120], the structure of the side chain of tyrosine at a specific position in CoV-M might have a marked effect on binding to S proteins [119].

The S protein sequence requirements for M-S interactions or VLP/virion incorporation have been reported in several CoVs. Experiments involving a chimeric S protein between MHV- and FIPV-S indicate that S-incorporation into VLPs or virions is determined only by the CT domain [104,121]. The CT domain of MHV-S proteins can be divided into a charge-rich region and highly conserved CRM, in which all cysteine residues are modified with palmitic acid (Figure 4a) [105,106,107,122]. Mutational analysis of the MHV-S CT domain has shown that the charge-rich region is important for S-incorporation into virions [123], while the palmitoylation of CRM is involved in fusion activity [105,106,107]. However, subsequent studies demonstrated that the palmitoylation of CRM also plays an important role in M-S interactions or/and S-incorporation, although the requirements for these functions differ (Table 3). The importance of palmitoylation for virion assembly was first indicated by an experiment using 2-bromopalmiate (2-BP), which inhibits protein palmitoylation. 2-BP treatment of MHV-infected cells caused under-palmitoylation of MHV-S proteins, which inhibited M-S interactions, resulting in reduced S-incorporation into virions [123]. Mutational analysis of the recombinant MHV virus revealed that cysteine residues at specific positions (CRM1B of JHM strains) have an effect on infectivity but not S-incorporation [123], while those of CRM2of A59 strain are required for M-S interactions and S-incorporation [124]. A later, more detailed mutational analysis of MHV-S (A59 strain), however, suggested that at least three cysteine residues in CRM, but not dependent on specific positions, are required for survival of a recombinant virus [125]. Since the reduced S-incorporation appears to be due to the lack of M-S interactions, the palmitoylation or cysteine residues within CRM of MHV-S proteins share two functions: M-S interactions and S-incorporation. In contrast, neither palmitoylation nor cysteine residues of SCoV-S proteins are required for M-S interaction, but are essential for S-incorporation into VLP (Table 3) [122,126]. Detailed mutational analysis identified only two cysteine residues at specific positions (CRM2B) to be required for VLP incorporation [122]. Recently, those roles of TGEV have been reported, revealing that palmitoylation of the S protein of the *alphacoronavirus* TGEV is essential for S-incorporation into VLPs irrespective of the position of the cysteine residues, but is dispensable for M-S interactions (Table 3) [127]. It is of interest that the palmitoylation (or cysteine residues) of MHV-S proteins share two functions, M-S interactions and S-incorporation, while SCoV- and TGEV-S proteins are required for S-incorporation but not for M-S interactions. This difference can be explained as follows. Hydrophobic palmitic acids might interact with or insert into the inner leaflet of the lipid bilayer and fix the orientation of CT domains to expose a specific face, which is required for M-S interactions. Since MHV-S proteins lacking retention and retrieval signals pass through a secretory pathway only once, they have an opportunity to come into contact with M proteins only at the budding site. If this specific face is not exposed at the time of contact, they do not interact with M proteins and are released to the cell surface, resulting in a strict requirement for palmitoylation in M-S interactions. In contrast, since TGEV- and SCoV-S proteins carrying these signals have several opportunities to come into contact with M proteins at the budding site, even though the specific face is not always exposed, it might be exposed occasionally, resulting in an interaction with M proteins in due time. Thus, palmitic acids are dispensable for the M-S interactions of TGEV- and SCoV-S proteins. On the other hand, palmitoylation is essential for the S-incorporation of all CoV-S proteins, although the position requirement is different. It is also possible that hydrophobic palmitic acids cause intra- or inter-molecular interactions that enable tight packing of the CT domain. As mentioned above, since the lattice-like matrix formed by lateral M-M interactions limits the space available for membrane-spanning S proteins, efficient S-incorporation is required for the tight packing or the formation of an appropriate CT domain to accommodate the endodomain. These studies demonstrate the various requirements of palmitoylation for M-S interactions and S incorporation.

**Table 3:** Summary of the roles of palmitoylation (cysteine residues) of several CoV-S proteins in M-S interaction and VLP/virion incorporation *.

Genus	Species	M-S Interaction	VLP/Virion Incorporation (or Infectivity **)	
			Palmitoylation	Position specificity
**Alpha CoV**	**TGEV**	Independent [127]	**Dependent** [127]	No specific position [127]
**Beta CoV**	**MHV**	**Dependent** [123,124]	**Dependent** [123,124]	**Specific position (CRM1B)** ** [123] **Specific position (CRM2)** [124] No specific position [125] **
	**SCoV**	**Independent** [122,126]	**Dependent** [122]	**Specific position (CRM2B)[122]**

* This table is a modification of a table in reference 120. ** The mutants show little or no infectivity, although S incorporation is not affected [123] or not studied [125].

The roles of CoV-S retention or retrieval signals in VLP or virion incorporation have not been investigated extensively, although their importance has been suggested. The contribution of two IBV-S retention signals to virus infection was evaluated using recombinant IBV. Recombinant IBV lacking an ER retrieval signal (KKxx) exhibited reduced growth but was viable, while the tyrosine signal of IBV-S is essential for survival of recombinant IBV [128]. A murine-adapted PEDV lacking an ER retrieval signal (KxHxx motif) showed a growth ability and S-incorporation into virions similar to those of the original PEDV [129]. Although the importance of ER retrieval signals has been reported when S proteins expressed independently. [38,114], in the whole virion ER retrieval signal of both IBV- and PEDV-S (KKxx and KxHxx motifs) had little effect on viral infection or S-incorporation. In contrast, the ER retrieval signals (KxHxx motif) of SCoV-S appeared to have a marked effect on S-incorporation into VLPs (unpublished data). These findings suggest that the requirement of the ER retrieval signal for S-incorporation differs among CoV-S proteins.

## 5. Conclusions and Perspective

Since virus assembly is a key event in the virus replication cycle, understanding virus assembly is important for controlling viral infection and developing antiviral drugs. Unlike other enveloped viruses, CoVs bud and assemble at the intracellular compartment ERGIC, and the M, E, and some S envelope proteins of CoV can be retained intracellularly. However, it is uncertain as to which proteins or factors control the site of budding, as many proteins pass through the ERGIC but accumulate at the Golgi complex beyond the budding site when expressed independently. In addition, since experimental conditions—such as the cell type or protein expression system used—likely affect their subcellular localization, the same protein can show different localization. For example, IBV-S and IBV-E were reported to be localized to the ER or/and ERGIC [38,93] or the Golgi complex [39,111], respectively, which complicated the interpretation of their subcellular localization. A likely candidate determinant of the budding site is the E protein, as it is essential for VLP formation, and MHV and IBV-E proteins accumulate at the ERGIC [51,93] and MHV-E alone promotes ERGIC membrane rearrangement [51]. Nevertheless, the E proteins are not essential for production of virions of some CoVs, such as MHV and SCoV [27,28].

The high degree of conservation of the Golgi-retention signal of three CoV-M proteins (MHV, IBV and SCoV-M) suggests their significant contribution to CoV replication, most likely in virion assembly (M incorporation). However, some M proteins are capable of reaching the cell surface, and, interestingly, a plasma-membrane-targeting signal has been identified in the SCoV-M protein, although its biological role remains unclear (Figure 3b). Biochemical studies clearly demonstrated that, as well as the presence of CoV-E proteins, lateral interactions among M proteins via multiple contact sites of overall domains are important for M-incorporation into VLPs or virions (Figure 3d). These results hypothesized that M-M proteins would be responsible for formation of the lattice-like dense matrix within the CoV envelope. This model could explain many possible mechanisms, such as the selectivity of other envelope proteins as well as RNPs, exclusion of foreign proteins and the driving force for budding [45,46]. However, a recent electron microscopy study did not detect a well-ordered rigid lattice structure in individual virions, showing instead loosely ordered M-M protein networks [101], suggesting that the lattice-like matrix structure formed by M-M interactions might be flexible and unstable, and so that the model might need some modifications. 

Two retention signals, ER retrieval and a tyrosine-based localization signals, of the CT domain of CoV-S have been proposed to mediate their intracellular retention. In the cases of TGEV and IBV-S proteins, which harbor both signals, there is little agreement as to which signal is the “true” functional retention signal (Table 2). The reason for the discrepancy might be the ambiguity of the two signals. The tyrosine-based localization signal (YxxF and YxxI motifs) seems to act as either a retention or internalization signal, depending on the experimental conditions. This feature also demonstrates that the functions of the tyrosine-based signal of other CoVs should not be assumed until experimental results are reported. On the other hand, the potency of the ER retrieval signal varies depending on the surrounding amino acid sequence. In addition, since the overexpression of S proteins likely results in saturation of the cellular machinery that recognizes the ER retrieval signal, even the S protein with potent ER retrieval signal, would likely not be retained intracellularly under this condition. These ambiguities make interpretation of their roles more complicated. Interestingly, the S proteins of most *alpha*- and *gammacoronaviruses* contain two potential signals, while *beta*- and *deltacoronaviruses* do not (SCoV has only a weak ER retrieval signal). In addition to differences in the roles of retention signals, recent studies have focused on the differences in the role of palmitoylation in M-S interactions and VLP/virion incorporation. The reason for the inherent differences in roles among CoVs remains unknown. 

Research to date has demonstrated that CoVs share a general mechanism of S and M protein incorporation into virions. However, it has also emphasized that the various CoVs have functional differences in retention signals and protein-interaction motifs of S and M proteins, which would be required for incorporation into virions. Although the differences of CoVs are thought to be acquired by evolutionary forces in adapting to optimal replication strategies, further studies are necessary to understand the importance of their similarities and differences in CoV evolution. 
